# A Generalized Gamma Mixture Model for Ultrasonic Tissue Characterization

**DOI:** 10.1155/2012/481923

**Published:** 2012-12-04

**Authors:** Gonzalo Vegas-Sanchez-Ferrero, Santiago Aja-Fernandez, Cesar Palencia, Marcos Martin-Fernandez

**Affiliations:** Laboratorio de Procesado de Imagen, ETSI Telecomunicación Edificio de las Nuevas Tecnologías, Campus Miguel Delibes s/n, Universidad de Valladolid, 47011 Valladolid, Spain

## Abstract

Several statistical models have been proposed in the literature to describe the behavior of speckles. Among them, the Nakagami distribution has proven to very accurately characterize the speckle behavior in tissues. However, it fails when describing the heavier tails caused by the impulsive response of a speckle. The Generalized Gamma (GG) distribution (which also generalizes the Nakagami distribution) was proposed to overcome these limitations. Despite the advantages of the distribution in terms of goodness of fitting, its main drawback is the lack of a closed-form maximum likelihood (ML) estimates. Thus, the calculation of its parameters becomes difficult and not attractive. In this work, we propose (1) a simple but robust methodology to estimate the ML parameters of GG distributions and (2) a Generalized Gama Mixture Model (GGMM). These mixture models are of great value in ultrasound imaging when the received signal is characterized by a different nature of tissues. We show that a better speckle characterization is achieved when using GG and GGMM rather than other state-of-the-art distributions and mixture models. Results showed the better performance of the GG distribution in characterizing the speckle of blood and myocardial tissue in ultrasonic images.

## 1. Introduction

Among the noninvasive imaging modalities, probably, the most widespread are the ultrasound imaging. The main reason of its success is that it provides a low-cost way to help diagnosing and can be used for many medical applications. However, ultrasonic (US) images are characterized by the presence of a peculiar granular pattern: the so-called *speckle*.

This term was adopted from the field of laser optics [1] in the early 1960s due to the similarity of the patterns between laser optics and ultrasonics. Although the nature of the speckle in US images stems from a different phenomenon, there still share some similarities. Both patterns come from the random interference of many coherent wave components reflected from different microscopic elements. In the case of US, the volume, the number of effective scatterers, and the acquisition process contribute to the formation of a speckle [2].

The analysis of backscattered echo from tissues needs a proper description of the ultrasonic signals. For this purpose, and due to the random nature of the speckle, several statistical models have been proposed in the literature. This characterization can be used either for segmentation [3], classification [4] purposes or for filtering the speckle itself [5–8]. The latter usually considers the speckle as an undesired consequence, since it degrades resolution and adds spatial noise to the image. Thus, filtering is commonly applied as a preprocessing step for further segmentation of regions of interest or to extract relevant measures for physiological analysis.

The statistical description of US signals provide an important information of the backscattered echo from tissues. The parameters of the statistical models allow identifying the features of tissues and provides important descriptors for classification. Some of the filtering algorithms relay on a Bayesian approach where an accurate statistical model becomes necessary. As a consequence, modeling the amplitude statistics of US signals has been a very active area.

Several statistical models have been proposed in the last decades. Probably the most wellknown is the Rayleigh model, which is a one-parameter distribution which describes the so-called fully formed (or developed) speckle. This probabilistic distribution describes the behavior of a speckle when a high number of effective scatterers are present in the resolution cell. However, real images show a deviation from this model, this non-Rayleigh behavior can be due to a small number of scatterers in the resolution cell or when there are some dominant components in the cell. The most commonly accepted distributions that try to model non-Rayleigh distributions are the Rice (fully resolved speckle), *K* (partially formed speckle), and Homodyned *K* (partially resolved speckle).

Although, those models are based on physical assumptions of the backscattering process, some other distributions have proven to provide a good performance on real images. This is the case of Gamma [9–11] and Nakagami [12] distributions. The first is proposed as a two-parameter distribution that describes the result of interpolated/filtered fully formed speckle [9] and also has shown good results in empirical tests among other distributions [10, 11]. The Nakagami distribution proposed by Shankar for the case US characterization [12] is also a two-parameter distribution which generalizes the Rayleigh distribution. This distribution was adopted from the models proposed to describe the statistics of the returned echo radar.

The capability of the Nakagami distribution to model the backscattering from tissues for fully resolved and fully formed speckle made it become the most commonly accepted model for tissue characterization. However, the tails of the probabilistic density functions of Nakagami, K, Rayleigh, or Gamma do not show the impulsive response of speckle which originate heavier tails. In order to describe this impulsive response, a generalized Nakagami distribution was proposed by Shankar in [13]. This is a three-parameter model which has shown a better behavior than the Nakagami or Rayleigh, an expected result since it is a generalization of the other models. However, the generalized Nakagami distribution does not have closed-form maximum likelihood estimates (MLE) and, thus, it makes their use difficult. Note that, though Shankar in [13] said that the MLE can be obtained, the equations used were based on the results from Stacy and Mihram [14], which were calculated by the methods of moments and they also expressed the difficulties of obtaining an MLE: “Closed expressions for solutions to the maximum likelihood equations are highly unlikely.” It is important to note that the results of [14] were obtained for the estimation of the Generalized Gamma (GG) distribution which is essentially the same as the generalized Nakagami distribution but with another parametrization.

The different nature of tissues is reflected in a different response of the speckle. Hence, a mixture model has shown to be a natural strategy for statistically describing the features of tissues. This approach has been previously used for segmentation purposes in the case of Nakagami mixture models (NMMs) by Destrempes et al. in [3], for classification with Rayleigh Mixture Models by Seabra et al. in [4], and for filtering with a mixture of Gamma and Gaussian Mixture Models in [8, 9]. All these approaches make use of the Expectation-Maximization (EM) [15] algorithm to calculate the parameters that better fit the empirical probability distribution function (PDF). This method is particularly useful when the MLE exists since it maximizes the expected value of the log-likelihood function with respect to the condition of the belonging to each tissue class for a given data.

The EM algorithm cannot be easily applied for the calculation of a Generalized Gamma Mixture Model (GGMM) without an MLE. However, some interesting results have been recently published on the calculation of the MLE of the Generalized Gamma which permit efficient computation of the GGMM.

The aim of this work is to revitalize the use of the Generalized Gamma distribution (also called, Generalized Nakagami Distribution) for tissue characterization. For this purpose, we present two main contributions: first, we propose a simple methodology to calculate the ML estimate which offers robust results comparing to the methods in the literature [14, 16, 17]. Second, two different methods were proposed for the calculation of the GGMM parameters. Both were developed by applying the EM method in the derivation of the proposed ML method. Results when comparing both methods to the GMM and NMM in real images showed the better fitting of the GGMM. The GGMM provides a posterior probability of belonging to each tissue class which can be of help for further filtering, segmenting, or classifying methods.

The rest of the paper is structured as follows. In Section 2.1, we introduce the distributions most commonly used for characterizing speckle of ultrasonic images. There, the GG distribution is motivated as a suitable generalization of the Gamma and Nakagami distributions which fail in describing the impulsive response of speckle. Then, in Section 2.2, we analyze the methods proposed in the literature for estimating the parameters of the GG distribution and a simple but robust method is proposed (Section 2.2.4). One of the advantages of this method is that it can be easily extended to estimate the parameters of a GGMM by means of the EM algorithm.  Section 2.3 is devoted to the extension of the ML method to obtain the parameters of the GGMM where two algorithms are proposed. The performance of the ML estimate derived in Section 2.2.4 is compared to other state-of-the-art methods in Section 3.1 for synthetic data and for real cases in Section 3.2. The performance of the GGMM is analyzed in Section 3.3, where the GGMM is compared to NMM and GMM. Finally, we propose some applications for the GGMM in Section 3.4. In Section 4, we conclude.

## 2. Materials and Methods

### 2.1. Statistical Models for Describing the Nature of Speckle

The formation of US images begins with the emission of a pulse packet which travels through the tissue. The backscattering produced by the scatterers in the resolution cell contribute to the change of the pulse shape according to the characteristics of the media, that is, the number of scatterers as well as their size [4, 9, 12].

The contribution of the backscattered echo, *s*(*t*), can be treated as a random walk due to the random location of the scatterers in the resolution cell [12]:

(1) $$\begin{array}{c} s\left(t\right)=\sum_{n=1}^{N}\alpha_{n}\cos\left(\omega_{0}t+\phi_{n}\right), \end{array}$$

where *ω*_0_ is the mean frequency of excitation and *N* is the number of effective scatterers in the resolution cell. The phases, *ϕ*_*n*_, are usually modeled as uniformly distributed in [0,2*π*] and the amplitude is usually considered to be Normal distributed.

The fully formed speckle model assumes a high number of scatterers, so the *Central Limit Theorem* applies and the backscattered echo can be expressed as

(2) $$\begin{array}{c} s\left(t\right)=X\cos\left(\omega_{0}t\right)+Y\sin\left(\omega_{0}t\right), \end{array}$$

where *X* and *Y* are zero mean identically distributed Gaussian distributions.

Then, the envelop of the backscattered signal echo, $R=\sqrt{X^{2}+Y^{2}}$ is Rayleigh distributed [1, 18]:

(3) $$\begin{array}{c} f_{R}\left(r\right)=\frac{r}{\sigma^{2}}e^{-r^{2}/2\sigma^{2}}u\left(r\right), \end{array}$$

where *u*(·) is the Heaviside step function defined as

(4) $$\begin{array}{c} u\left(x\right)=\{\begin{array}{cc} 0, & x<0\\ 1, & x\geq 0. \end{array} \end{array}$$

Under the assumption of a high number of effective scatterers but with the presence of resolvable structures in the resolution cell (specular component, *C*), *X* and *Y* become nonzero Gaussian distributions. The envelop does no longer follow a Rayleigh distribution but a Rician one [18]:

(5) $$\begin{array}{c} f_{R}\left(r\right)=\frac{r}{\sigma^{2}}e^{-\left(r^{2}+C^{2}\right)/2\sigma^{2}}I_{0}\left(\frac{rC}{\sigma^{2}}\right)u\left(r\right), \end{array}$$

where *I*_0_(·) is the modified Bessel function of first kind.

When the number of scatterers decreases and the Central Limit Theorem cannot be applied, more complicated distributions are proposed to model the distribution of the envelope. Concretely, the *K* distribution models the case when the number of scatterers is a random variable itself, which is modeled as a Poisson whose local mean is Gamma distributed, this is equivalent to consider *σ* as gamma distributed [2]:

(6) $$\begin{array}{c} f_{R}\left(r\;|\;\sigma \right)=\frac{r}{\sigma^{2}}e^{-r^{2}/2\sigma^{2}}u\left(r\right),\\ f_{\sigma }\left(\sigma \right)=\frac{1}{2b^{2}}\frac{1}{\Gamma \left(\nu +1\right)}\left(\frac{\sigma }{2b^{2}}\right)e^{-\sigma /2b^{2}}u\left(\sigma \right), \end{array}$$

so, the PDF of *R* is

(7) fR(r)=∫fR(r ∣ σ)fσ(σ)dσ=2bΓ(ν+1)(σ2b2)ν+1Kν(rb)u(r),

where *K*_*ν*_(·) is the modified Bessel function of the second kind.

A generalization of the previous models appears when a specular component is considered and the number of scatterers, *N*, follows a negative binomial distribution. This is the case of the homodyned-K distribution [19]:

(8) $$\begin{array}{c} f_{R}\left(r\right)=r\left(\int \frac{x}{1+x^{2}\sigma^{2}/2\nu }J_{0}\left(xC\right)J_{0}\left(xr\right)dx\right)u\left(r\right). \end{array}$$

This PDF has no closed expression and this limits its use.

On a completely different approach, Shankar in [12] proposed a Nakagami distribution as a “simpler universal model for tissue characterization.” Unlike the previously reviewed models, the Nakagami is not based on physical arguments or on a bottom-up modeling of the scattering process. However, it has empirically shown a better performance than the Rayleigh and Rice distributions.

The Nakagami PDF is as follows:

(9) $$\begin{array}{c} f_{R}\left(r\right)=\frac{2m^{m}r^{2m-1}}{\Gamma \left(m\right){\left(2\Omega \right)}^{m}}e^{-(m/2\Omega )r^{2}}u\left(r\right). \end{array}$$

This distribution offers good properties to describe the backscattered echo: the Rayleigh distribution is a particular case of the Nakagami (*m* = 1) and, additionally, when *m* > 1 is similar to the Rice distribution. However, this distribution has some limitations. The Nakagami model cannot fit the heavier tails of the empirical PDFs due to the impulsive nature of scatterers [13].

In order to describe the impulsive response of scatterers, Shankar proposed in [13] a generalized Nakagami distribution which is essentially the same as a Generalized Gamma distribution [14]. However, this distribution presents some difficulties in the estimation of its parameters, since there are no closed equations for the ML estimates.

In the next section, we describe some methods that have been used in the literature with special attention to methods that provide an ML estimate of the GG parameters. Additionally, we propose a simple method to calculate the ML estimates of the parameters. The results obtained in the derivation of this ML method provide the foundations for the development of the Generalized Gamma Mixture Model, which is the main contribution of this work.

### 2.2. Estimation of Parameters of the Generalized Gamma

#### 2.2.1. Moments Method

This method was proposed by Stacy in [14]. For the derivation of the method, the following parametrization was adopted:

(10) $$\begin{array}{c} f\left(x\;|\;a,\nu ,p\right)=p\frac{x^{p\nu -1}}{a^{p\nu }\Gamma \left(\nu \right)}e^{-{\left(x/a\right)}^{p}}u\left(x\right), \end{array}$$

where the parameters (*a*, *ν*, *p*) are all positive.

This is the definition of the GG distribution hereafter. For a given *p* > 0, all moments *E*{*X* ^*r*^} exist.

Now, let *Z* be the random variable (RV) defined as

(11) $$\begin{array}{c} Z=\log{\left(\frac{X}{a}\right)}^{p}=p\left(\log\left(X\right)-\log\left(a\right)\right). \end{array}$$

For this RV, the central moments, *μ*_*r*_(·), of rth order are

(12) $$\begin{array}{c} \mu_{r}\left(Z\right)=p^{r}\mu_{r}\left(\log X\right). \end{array}$$

Additionally, it is easy to show that, given a RV, *X*, which follows a GG distribution (*X* ~ *GG*(*a*, *ν*, *p*)), the following properties hold:

(13) $$\begin{array}{c} kX\sim GG\left(ka,\nu ,p\right),\;k>0\\ X^{m}\sim GG\left(a^{m},\nu ,\frac{p}{m}\right),\;m\neq 0. \end{array}$$

So, a new RV *Z* can be defined as *Z* = log⁡(*X*/*a*)^*p*^ where (*X*/*a*)^*p*^ follows a Gamma distribution of parameter *ν*. Hence, the log-transformed distribution of the Gamma RV is the following:

(14) $$\begin{array}{c} f_{Z}\left(z\right)=f_{GG}\left(e^{z}\;|\;1,\nu ,1\right)e^{z}=\frac{1}{\Gamma \left(\nu \right)}\exp\left(\nu z-\exp\left(z\right)\right), \end{array}$$

where *z* ∈ ℝ.

The moment generating function of *Z* is easily calculated as *E*{*e* ^*tZ*^} = (*t* + *ν*)/*ν*. Where *E*{·} is the expectation operator. So, the *r*th moment of *Z* is the following:

(15) $$\begin{array}{c} E\left\{Z^{r}\right\}=\frac{\Gamma^{\left(r\right)}\left(\nu \right)}{\Gamma \left(\nu \right)}=\Psi^{\left(r\right)}\left(\nu \right), \end{array}$$

where Ψ^(*r*)^ is the *polygamma* function defined as

(16) $$\begin{array}{c} \Psi^{\left(r\right)}\left(x\right)=\frac{d^{m+1}}{dx}\log\Gamma \left(x\right). \end{array}$$

Finally, the three first central moments are defined as:

(17) $$\begin{array}{c} pE\left\{\log X-\log a\right\}=\Psi^{\left(0\right)},\\ p^{2}\mu_{2}\left(\log X\right)=\Psi^{\left(1\right)},\\ p^{3}\mu_{3}\left(\log X\right)=\Psi^{\left(2\right)}. \end{array}$$

These equations can be used to estimate the parameters of the *GG*(*a*, *ν*, *p*); $\hat{a},\hat{\nu },\hat{p}$, by approximating the moments by means of the sample moments:

(18) $$\begin{array}{c} \hat{a}=\exp\left(\overset{-}{y}-\Psi^{\left(0\right)}\left(\hat{\nu }\right)\right),\\ \hat{p}=-\mathrm{sign}\left(g_{y}\right)\frac{\sqrt{\Psi^{\left(1\right)}\left(\hat{\nu }\right)}}{S_{y}},\\ -\left|g_{y}\right|=\frac{\Psi^{\left(2\right)}\left(\hat{\nu }\right)}{{\left(\Psi^{\left(1\right)}\left(\hat{\nu }\right)\right)}^{3/2}}, \end{array}$$

where $\overset{-}{y}=\left(1/N\right)\sum_{i=1}^{N}\log x_{i}$, with {*x*_*i*_}_*i*=1_^*N*^ the set of samples of *X*; *S*_*y*_^2^ is the sample variance of {*y*_*i*_}_*i*=1_^*N*^ = {log⁡*x*_*i*_}_*i*=1_^*N*^, and *g*_*y*_ its sample skewness.

The estimates are derived by means of calculating the value $\hat{\nu }$ from the last equation of (18). So, a numerical calculation needs to be performed. In the original article [14], Stacy and Mihran provided a graph representing Ψ^(2)^(*ν*)/(Ψ^(1)^(*ν*))^3/2^ for a range *ν* ∈ [0.1,5].

This method, though provides a quite straight-forward calculation of the parameters, can provide estimates which are outside the parameter space. Yet, it is highly sensitive to the number of samples.

#### 2.2.2. Heuristic Approaches

In order to avoid the problems associated to the moments method, some heuristic methods have been proposed in the literature. As examples, Gomes et al. [16] proposed an iterative method which evaluates the best performance of the *χ* ^2^ goodness-of-fit test for a fixed *p* (see the parametrization of (10)). The parameters of the transformed samples *Y* = *X* ^*p*^, which are Gamma distributed, were calculated by the moments method. At the end of the loop, the set of parameters with least *P* value is chosen.

This method presents some shortcomings. First, the parameters of the Gamma distributed data were calculated by the moments method, so the problems associated to the moments method are not circumvent. However, even if a good estimate is calculated, the *χ* ^2^ goodness-of-fit test depends on the calculation of the estimated PDF which strongly depends on the number of bins considered and the assumption of a sample with sufficient large size.

Other heuristic method is the one presented by Wingo in [20]. This method, based on the one proposed by Hager and Bain [21], tries to solve the maximum likelihood equations for the GG distribution. The log-likelihood, *ℒℒ*, of a RV *X* ~ *GG*(*a*, *ν*, *p*) for the parametrization presented in (10) is

(19) ℒℒ(a,ν,p ∣ x)=log⁡((papνΓ(ν))n∏i=1nxipν−1e−∑i=1n(xi/a)p)=nlog⁡(p)−npνlog⁡(a)−nlog⁡(Γ(ν))+(pν−1)∑i=1nlog⁡xi−∑i=1n(xia)p,

where **x** = {*x*_*i*_}_*i*=1_^*n*^ is the set of samples.

Now, calculating the derivatives with respect to the parameters and setting it equal to zero, one can obtain the ML equations:

(20) $$\begin{array}{c} a^{p}=\frac{1}{n\nu }\sum_{i=1}^{n}x_{i}^{p},\\ p\sum_{i=1}^{n}\log\left(\frac{x_{i}}{a}\right)-n\Psi \left(\nu \right)=0,\\ \frac{n}{p}+\nu \sum_{i=1}^{n}\log\left(\frac{x_{i}}{a}\right)-\sum_{i=1}^{n}{\left(\frac{x_{i}}{a}\right)}^{p}\log\left(\frac{x_{i}}{a}\right)=0, \end{array}$$

where Ψ(*x*) ≡ Ψ^(0)^(*x*) = Γ′(*x*)/Γ(*x*).

This system of equations can be reduced to a single nonlinear equation with *p* as the single unknown:

(21) $$\begin{array}{c} -\Psi \left(\nu \right)+\frac{p}{n}\sum_{i=1}^{n}\log\left(x_{i}\right)-\log\left(\sum_{i=1}^{n}x_{i}^{p}\right)+\log\left(n\nu \right)=0, \end{array}$$

where

(22) $$\begin{array}{c} \nu =-{\left(\frac{p}{n}\sum_{i=1}^{n}\log\left(x_{i}\right)-p\frac{\sum_{i=1}^{n}x_{i}^{p}\log\left(x_{i}\right)}{\sum_{i=1}^{n}x_{i}^{p}}\right)}^{-1},\\ a={\left(\frac{1}{n\nu }\sum_{i=1}^{n}x_{i}^{p}\right)}^{1/p}. \end{array}$$

So, the problem is reduced to calculate *p* from (21). Some authors reported the difficulty of solving this equation with the conventional numerical methods such as Newton-Raphson [21] and conclude that the MLE may not exist.

In [20], the author faced the problem by analyzing the effect of inappropriate zero finding algorithms. So, an heuristic method for isolating roots of a general scalar nonlinear equation was proposed. This method makes use of the root-isolation technique proposed in [22], which uses only function values to isolate the roots in a compact interval of the real line.

Though this method can provide an ML estimate of the parameter by solving (21), it has to heuristically divide the intervals where *p* is searched and calculate whether a root is in it or not by means of the mean value and variance of the function in each of the intervals, so many evaluations of the function are required.

#### 2.2.3. ML Approach

A very interesting analysis was recently published by Noufaily and Jones in [17], where an iterative approach is proposed to solve the likelihood equations, (20), in a way that the individual equations are uniquely solvable. This result provides a very promising technique for calculating the MLE parameters of the GG.

In that work, the log-likelihood equations were calculated following the re-parametrization proposed in [23]. Concretely, for a RV *X* which is distributed, *X* ~ *GG*(*a*, *ν*, *p*), the new RV *Y* = log⁡*X* is calculated, whose PDF is the following:

(23) fY(y)=pΓ(ν)eypνapνexp⁡(−eypap)=νν−1/2σΓ(ν)exp⁡(νw−νew/ν),

where *y* ∈ ℝ, *w* = (*y* − *μ*)/*σ*, $\sigma =1/p\sqrt{\nu }$ and *μ* = log⁡(*a*) + (1/*p*)log⁡(*ν*).

So, in the end, the following equations have to be solved:

(24) $$\begin{array}{c} \mu =\sigma \sqrt{\nu }\log S_{0}, \end{array}$$

(25) $$\begin{array}{c} R\left(\sigma \right)\equiv \frac{S_{0}}{S_{1}}-\overset{-}{Y}-\frac{\sigma }{\sqrt{\nu }}=0, \end{array}$$

(26) $$\begin{array}{c} T\left(\nu \right)\equiv \log\left(\nu \right)-\Psi \left(\nu \right)-\frac{L}{\sqrt{\nu }}=0, \end{array}$$

where $L=\left(\mu -\overset{-}{Y}\right)/\sigma$ and $S_{j}=\left(1/n\right)\sum_{i=1}^{n}y_{i}^{j}\exp\left(y_{i}/\sigma \sqrt{\nu }\right)$.

The important result of [17] is the demonstration that both (25) and (26) are well behaved with unique solutions in *σ* and *ν*, respectively. So, an iterative method can be developed to calculate $\hat{\nu }$ by (26) from an initial guest of the parameters and then $\hat{\sigma }$ by solving (25). Finally, $\hat{\mu }$ is calculated by replacing the previous estimates in (24). These estimates can be used to calculate a new *L* to compute the new log-likelihood function. By repeating these steps until a desired accuracy, the estimates are achieved [23].

This method provides a fundamental result about the behavior of the log-likelihood equations, and guarantees their solution. However, the method does not provide any proof concerning its convergence or the uniqueness of the ML. Yet, this method needs to solve two nonlinear equations by numerical techniques, whereas the method proposed by Wingo in [20], previously described, only needs to solve a linear equation.

#### 2.2.4. The Proposed Approach

We propose a simple but efficient method to calculate the ML estimates of the GG distribution. The main advantage of the method is that it can be easily implemented and has the same properties of the method of [17], that is, the equation to solve are well behaved with unique solution. Additionally, the method just needs the calculation of just one non-linear equation and, thus, the computing time is considerably reduced.

The method consists in transforming the RV, *X* ~ *GG*(*a*, *ν*, *p*) by the following transformation *Y* = *X* ^*p*_0_^ where *p*_0_ is a positive real number. So, the new PDF of *Y* is the following:

(27) $$\begin{array}{c} f_{Y}\left(y\right)=\frac{p}{p_{0}}\frac{y^{(p/p_{0})\nu -1}}{a^{p\nu }\Gamma \left(\nu \right)}\exp\left(-\frac{y^{p/p_{0}}}{a^{p}}\right)u\left(y\right). \end{array}$$

Note that this PDF follows a Gamma PDF when *p*_0_ = *p*. Hence, a reasonable way to find the *P* value is to find the value of *p*_0_ that maximizes the likelihood of the GG distribution and also maximizes the Gamma distributed RV *Y* = *X* ^*p*_0_^.

In order to see if this method provides a proper solution, we first demonstrate that the ML estimate of the parameters of the new random variable *Y* also maximizes the likelihood of the GG distribution when *p*_0_ = *p*.

First, we calculate the ML estimates of the parameters of (27) for *p*_0_ = *p*, whose log-likelihood is the following:

(28) ℒℒY=−npνlog⁡(a)−nlog⁡(Γ(ν))+(ν−1)∑i=1nlog⁡(yi)−∑i=1nyiap.

The maximum with respect to the parameter *a* is easily calculated by taking the derivative with respect to the *a* and setting it equal to zero:

(29) $$\begin{array}{c} a_{0}^{p}=\frac{1}{n\nu }\sum_{i=1}^{n}y_{i}. \end{array}$$

Finally, (28) can be maximized with respect to *ν* by introducing the value of *a*_0_:

(30) $$\begin{array}{c} \log\left(\nu \right)-\Psi \left(\nu \right)=\log\left(\frac{1}{n}\sum_{i=1}^{n}y_{i}\right)-\frac{1}{n}\sum_{i=1}^{n}\log\left(y_{i}\right). \end{array}$$

Now, by introducing *a*_0_ in the log-likelihood function of the GG distribution, (19):

(31) ℒℒX=nlog⁡(p)−nυlog⁡(1nυ∑i=1nyi)−nlog⁡(Γ(ν))+(ν−1p)∑i=1nlog⁡(yi)−nν.

Now, by maximizing with respect to *ν*, we obtain the following equation:

(32) ∂ℒℒX∂ν≡−nlog⁡(1n∑i=1nyi)+nlog⁡(ν)+∑i=1nlog⁡(yi)−nΨ(ν)=0,

and finally, reordering terms, we obtain the same equation for which *ν*_0_ is also a solution.

This result guarantees that there exists always a solution for the ML estimate of the GG distribution ($\hat{a}$, $\hat{\nu }$, $\hat{p}$) and the parameters $\hat{a}$ and $\hat{\nu }$ are those obtained for the ML estimate for the transformed RV $Y=X^{\hat{p}}$. Hence, there is always a solution for the MLE for a GG.

Additionally, since the MLE of a Gamma distribution always exist for whatever positive *y*_*i*_ values ((30) is well behaved), the problem is reduced to finding the value *p* that maximizes *ℒℒ*_*X*_ among the ones that maximize *ℒℒ*_*Y*_.

The search method for *p* was implemented by the Nelder-Mead method [24] while the Brent's algorithm was applied for calculating *ν* [25].

This method does not demonstrate the uniqueness of *p* as did not any of the methods in the literature. However, in our experience, we agree with Noufaily and Jones [17] that the global maximum of the *ℒℒ*_*X*_ appears to be distant to any other local maximum.

The main advantage of the method here proposed is that it is easy to implement and only one non linear equation has to be solved, whereas the method of [17] needs to solve two non-linear equations in each iteration and [20] method needs several calculations of non-linear equations for each interval considered for the isolation root technique.

### 2.3. Generalized Gamma Mixture Model

An additional advantage of the proposed method for the calculation of the MLE parameters for the GG distribution is that it can be easily adapted for the calculation of the parameters of GG Mixture Models (GGMM).

There were some attempts in the literature to obtain the parameters of a GGMM. Concretely, in [26], they calculated the GGMM by means of the Nelder-Mead and Gradient descent methods for maximizing the log-likelihood. However, that method is strongly sensitive to the number of mixtures since it is just a direct optimization of the log-likelihood score equations of the mixture model.

In this section, we derive the GGMM by applying the Expectation-Maximization methodology [15] and combining them with the method used to calculate the MLE of the GG distribution.

Let *X* = {*x*_*i*_}, 1 ≥ *i* ≥ *N* be a set of samples. These samples are considered to be independent and identically distributed (IID) RVs. Now, the GGMM considers that these samples result from the contributions of *J* distributions:

(33) $$\begin{array}{c} p\left(x_{i}\;|\;\Theta \right)=\sum_{j=1}^{J}\pi_{j}f_{X}\left(x_{i}\;|\;\Phi_{j}\right), \end{array}$$

where Φ is a vector of the parameters of the GGMM (*π*_1_,…, *π*_*J*_, Θ_1_,…, Θ_*J*_) and Θ_*j*_ are the parameters of the PDF (in our case the parameters of the GG, represented as *a*_*i*_, *ν*_*j*_, *p*_*j*_).

The joint distribution of IID samples is given by

(34) $$\begin{array}{c} p\left(X\;|\;\Theta \right)=\prod_{i=1}^{N}p\left(x_{i}\;|\;\Theta \right). \end{array}$$

The EM is applied here to maximize the log-likelihood function when some hidden discrete random variables *Z* = {*Z*_*i*_} are introduced into the model. These RVs take values in {1,…, *J*} and indicate the class for which each sample *x*_*i*_ belongs.

Now, defining Θ^(*n*)^ as an estimate of the parameters of the mixture in the *n*th iteration, the expectation step is performed by calculating the expected value of the log-likelihood *ℒℒ*(Θ | *X*, *Z*):

(35) $$\begin{array}{c} 𝒬\left(\Theta \;|\;\Theta^{\left(n\right)}\right)=E_{Z\;|\;\Theta^{\left(n\right)}}\left\{ℒℒ\left(\Theta \;|\;X,Z\right)\right\}. \end{array}$$

In the maximization step, the new estimate Θ^(*n*)^ is obtained by maximizing the expectation of the log-likelihood function 𝒬(Θ | Θ^(*n*)^). These steps are iterated until a stop criterion such as 𝒬(Θ | Θ^(*n*+1)^) − 𝒬(Θ | Θ^(*n*)^) < Tol for some preestablished tolerance (Tol) is reached.

The application of the EM algorithm for estimating the parameters of mixture models has been applied for several distributions, see, for example, [15, 27]. However, to the best of our knowledge, this is the first time a mixture model is presented for GG distributions.

In order to derive the estimates of the parameters in each iteration, we first define the joint distribution of IID samples *X* and the hidden random variables, *Z* as

(36) $$\begin{array}{c} p\left(X,Z\;|\;\Theta \right)=\prod_{i=1}^{N}p\left(x_{i},z_{i}\;|\;\Theta \right), \end{array}$$

where *p*(*x*_*i*_, *z*_*i*_Θ) = *p*(*x*_*i*_ *z*_*i*_, Θ)*p*(*z*_*i*_Θ).

Now, the log-likelihood function can be defined in the following way:

(37) ℒℒ(Θ ∣ X,Z)  =log⁡(p(X,Z ∣ Θ))=∑i=1Nlog⁡p(xi,zi ∣ Θ)=∑i=1Nlog⁡p(xi ∣ zi,Θ)+∑i=1Nlog⁡p(zi ∣ Θ)=∑i=1Nlog⁡fX(xi ∣ zi,Θ)+∑i=1Nlog⁡πzi.

The expectation of the log-likelihood function with respect to the hidden RVs when data {*x*_*i*_} and the previous estimate Θ^(*n*)^ are known as:

(38) 𝒬(Θ ∣ Θ(n))=EZ ∣ Θ(n){ℒℒ(Θ ∣ X,Z)}=∑i=1NEZ ∣ Θ(n),xi{log⁡fX(xi ∣ Θ)+log⁡p(zi ∣ Θ)}=∑i=1N‍ ∑j=1Jp(Zi=j ∣ xi,Θ(n))  ×(log⁡fX(xi ∣ Θzi)+log⁡πj),

where *π*_*j*_ = *p*(*Z*_*i*_ = *j* | Θ) is the probability of *x*_*i*_ to belong to the class *j*.

The probability *p*(*Z*_*i*_ = *j* | *x*_*i*_, Θ^(*n*)^) can be calculated by applying the Bayes theorem as

(39) $$\begin{array}{c} p\left(Z_{i}=j\;|\;x_{i},\Theta^{\left(n\right)}\right)=\frac{f_{X}\left(x_{i}\;|\;\Theta^{\left(n\right)}\right)p\left(Z_{i}=j\;|\;\Theta^{\left(n\right)}\right)}{p\left(x_{i}\;|\;\Theta^{\left(n\right)}\right)}. \end{array}$$

Note that (37) is composed of two terms, so the maximization step can be done to each term independently. For the term depending on the *π*_*j*_ some constraints have to be considered since they must hold ∑_*j*=1_^*J*^ *π*_*j*_ = 1. An optimization via Lagrange's multipliers can be done in a straightforward way and they guarantee a necessary condition for optimality in this problem. The new Lagrange function with *λ* as the Lagrange multiplier is the following:

(40) $$\begin{array}{c} \Lambda \left(\pi ,\lambda \right)=\sum_{i=1}^{N}\;‍\sum_{j=1}^{J}\gamma_{i,j}\log\pi_{j}+\lambda \left(\sum_{j=1}^{J}\pi_{j}-1\right), \end{array}$$

where we introduced *γ*_*i*,*j*_ = *p*(*Z*_*i*_ = *j* | *x*_*i*_, Θ^(*n*)^) to simplify notation.

Now, calculating the derivative with respect to each *π*_*j*_ and setting it equal to 0, the following expression is derived:

(41) $$\begin{array}{c} \sum_{i=1}^{N}\gamma_{i,j}=-\lambda {\hat{\pi }}_{j}. \end{array}$$

By summing both terms of the equation over *j*, we obtain *λ* = −∑_*i*=1_^*N*^∑_*j*=1_^*J*^ *γ*_*i*,*j*_ = −*N* and the estimates for the parameters *π*_*j*_ that maximize the Lagrange function (and the likelihood function) are

(42) $$\begin{array}{c} {\hat{\pi }}_{j}=\frac{1}{N}\sum_{i=1}^{N}\gamma_{i,j}=\frac{1}{N}\sum_{i=1}^{N}p\left(Z_{i}=j\;|\;x_{i},\Theta^{\left(n\right)}\right). \end{array}$$

For the calculation of the maximum of (37) with respect to Θ_*j*_ = (*a*_*j*_, *ν*_*j*_, *p*_*j*_), we first calculate the derivative with respect to *a*_*j*_:

(43) $$\begin{array}{c} \frac{\partial }{\partial a_{j}}\left\{\sum_{i=1}^{N}\;\sum_{j=1}^{J}\gamma_{i,j}\log f_{X}\left(x_{i}\;|\;\Theta_{j}\right)\right\}=0, \end{array}$$

where the log-likelihood of *p*(*x*_*i*_ | Θ_*j*_) is the one described in (19) for one sample *x*_*i*_:

(44) log⁡fX(xi ∣ Θj)=log⁡p−pνlog⁡(a)−log⁡Γ(ν)+(pν−1)log⁡xi−(xia)p.

The result is

(45) $$\begin{array}{c} {\hat{a}}^{p_{j}}=\frac{\sum_{i=1}^{N}\gamma_{i,j}x_{i}^{p_{j}}}{\nu_{j}\sum_{i=1}^{N}\gamma_{i,j}}. \end{array}$$

Now, plugging (45) into (37) and deriving with respect to *ν*_*j*_ and setting it equal to 0

(46) $$\begin{array}{c} \frac{\partial }{\partial \nu_{j}}\left\{\sum_{i=1}^{N}\;\sum_{j=1}^{J}\gamma_{i,j}\log f_{X}\left(x_{i}\;|\;\frac{\sum_{i=1}^{N}\gamma_{i,j}x_{i}^{p_{j}}}{\nu_{j}\sum_{i=1}^{N}\gamma_{i,j}},\nu_{j},p_{j}\right)\right\}=0. \end{array}$$

It results in the following equality:

(47) $$\begin{array}{c} \log\left(\nu_{j}\right)-\Psi \left(\nu_{j}\right)=\log\left(\frac{\sum_{i=1}^{N}\gamma_{i,j}x_{i}^{p}}{\sum_{i=1}^{N}\gamma_{i,j}}\right)+\frac{\sum_{i=1}^{N}\gamma_{i,j}\log\left(x_{i}^{p_{j}}\right)}{\sum_{i=1}^{N}\gamma_{i,j}}. \end{array}$$

Note that (47) is essentially the same as (30), which is well behaved and always has a unique solution. Thus, this nonlinear equation can be solved by numerical methods in the same way as was performed in the MLE of the GG parameters. In our case, we also used the Brent's algorithm [25].

The interval where the Brent's algorithm is performed can be derived by means of the following property:

(48) $$\begin{array}{c} \frac{1}{2\nu_{j}}<\log\left(\nu_{j}\right)-\Psi \left(\nu_{j}\right)<\frac{1}{\nu_{j}}. \end{array}$$

So, the desired value of ${\hat{v}}_{j}$ in the interval

(49) $$\begin{array}{c} \frac{1}{2A}<{\hat{\nu }}_{j}<\frac{1}{A}, \end{array}$$

where

(50) $$\begin{array}{c} A=\log\left(\frac{\sum_{i=1}^{N}\gamma_{i,j}x_{i}^{p_{j}}}{\sum_{i=1}^{N}\gamma_{i,j}}\right)+\frac{\sum_{i=1}^{N}\gamma_{i,j}\log\left(x_{i}^{p_{j}}\right)}{\sum_{i=1}^{N}\gamma_{i,j}}. \end{array}$$

This property can be found in [28] and was also used in [17] for the calculation of the ML estimates of the GG.

Now, the problem can be stated in the same way as was done for the ML estimate proposed in Section 2.2.4. We are interested in the parameter *p*_*j*_ which maximizes the likelihood for the component *j* ∈ [1, *J*]. So, for each *p*_*j*_, (45) and (47) provide the estimate of *a*_*j*_ and *ν*_*j*_, respectively. By applying the Nelder-Mead algorithm to maximize the log-likelihood function for each component *j*, as was done for the ML estimates in Section 2.2.4, one can obtain the desired ML estimates. We will refer to this method as the GGMM_1_ method.

It is important to note that the parameter estimates can be also solved by extending the ML method of [17]. For this purpose, the parametrization proposed by Lawless [23] can be applied to the mixture model as was explained in Section 2.2.3.

The log-likelihood equations to be solved are completely equivalent to (45) and (47) due to the invariance of the ML estimates to the transformation *Y* = log⁡(*X*). However, Lawless' parametrization allows us to extend the results of [17] to the case of GGMM. For the sake of clarity, we rewrite the parametrization:

(51) $$\begin{array}{c} \sigma_{j}=\frac{1}{p_{j}\sqrt{\nu_{j}}},\\ \mu_{j}=\log\left(a_{j}\right)+\frac{1}{p_{j}}\log\left(\nu_{j}\right),\\ k_{j}=\nu_{j}. \end{array}$$

With this parametrization, (45) becomes

(52) $$\begin{array}{c} {\hat{\mu }}_{j}=\sqrt{k_{j}}\sigma_{j}\log\left({\tilde{S}}_{0}\right), \end{array}$$

where

(53) $$\begin{array}{c} {\tilde{S}}_{r}=\frac{\sum_{i=1}^{N}\gamma_{i,j}y_{i}^{r}e^{\left(y_{i}/\sigma_{j}\sqrt{k_{j}}\right)}}{\sum_{i=1}^{N}\gamma_{i,j}}. \end{array}$$

So, in the case of the parameter *σ*_*j*_ which maximizes the log-likelihood of *Y* = log⁡(*X*):

(54) $$\begin{array}{c} \frac{\partial }{\partial \sigma_{j}}\left\{\sum_{i=1}^{N}\;\sum_{j=1}^{J}\gamma_{i,j}\log f_{Y}\left(y_{i}\;|\;{\hat{\mu }}_{j},\sigma_{j},k_{j}\right)\right\}=0. \end{array}$$

It results in

(55) $$\begin{array}{c} \frac{{\tilde{S}}_{1}}{{\tilde{S}}_{0}}-\frac{\sigma_{j}}{\sqrt{k_{j}}}-\frac{\sum_{i=1}^{N}\gamma_{i,j}y_{i}}{\sum_{i=1}^{N}\gamma_{i,j}}=0. \end{array}$$

This equation is well behaved and all the theoretical demonstrations obtained in [17] still hold: it is monotone decreasing and, when lim⁡*σ*_*j*_ → 0, the function takes the value

(56) $$\begin{array}{c} y_{\max}-\frac{\sum_{i=1}^{N}\gamma_{i,j}y_{i}}{\sum_{i=1}^{N}\gamma_{i,j}}>0. \end{array}$$

As a conclusion, (55) has always a positive solution for any *μ*_*j*_ and *k*_*j*_. Additionally, due to the invariance of the ML estimates for the transformation *Y* = log⁡(*X*), there always exist a *p*_*j*_ for any *a*_*j*_ and *ν*_*j*_.

The solution is in the interval

(57) $$\begin{array}{c} 0<{\hat{\sigma }}_{j}<\sqrt{k_{j}}\left(y_{\max}-\frac{\sum_{i=1}^{N}\gamma_{i,j}y_{i}}{\sum_{i=1}^{N}\gamma_{i,j}}\right). \end{array}$$

So, the value can be calculated by any numerical method. We used here also the Brent's algorithm.

So, finally, from an initial guess of *p*_*j*_ one can calculate *k*_*j*_ ≡ *ν*_*j*_ from (47) and then use it to calculate the estimate of *σ*_*j*_ from (55), in an iterative way until a desired tolerance is reached.

This methodology generalizes the proposed method of [17] for the case of GGMM and we will refer to it as the GGMM_2_ method.

### 2.4. Implementation Generalized Gamma Mixture Model

In this section, we detail the implementation of both of the proposed methods for the GGMM.

In Algorithm 1, the Nelder-Mead method [24] was used for the calculation of *p*_*j*_^(*n*)^ and the Brent's algorithm [25] for *ν*_*j*_^(*n*)^ in the interval given in (49).

In the case of Algorithm 2, the Brent's algorithm [25] was used for calculating *σ* and *k* in the intervals of (57) and (49), respectively.

The computational complexity of the previous GGMM methods when compared to the calculation of a simple GG depends on the number of components, *J*, assumed by the model. In each iteration of the EM algorithm, the expected parameters of each component have to be calculated. So, if the time consumed to estimate a GG is *T*, the calculation of the expected parameters of the mixture is *J* × *T*.

When other mixture models such as RMM, NMM, and GMM are considered, the complexity of the EM method is similar to the GGMM. Note that both the GMM and the NMM need to solve a non-linear equation similar to (47) so the consumed time of the solution is the same. The additional cost of calculating the GGMM parameters is due to the calculation of the estimate of the parameter *p*_*j*_^(*n*)^. If we define the time to solve (47) and (45) as *T*_1_, and *T*_2_ as the time consumed for solving *p*_*j*_^(*n*)^, the computational time for a simple GG (*T*_GG_) and a GGMM of *J* components (*T*_GGMM_) would be

(58) $$\begin{array}{c} T_{\text{GG}}=T_{1}+T_{2},\\ T_{\text{GGMM}}=J\cdot \left(T_{1}+T_{2}\right). \end{array}$$

The estimated times in a Matlab (R2011a) implementation running in an ASUS G53SW laptop (Intel Core i7 2630QM Processor, 2.2 GHz, 8 GB RAM) were *T*_1_ = 1.637 ms and *T*_2_ = 0.2056 s.

## 3. Results and Discussion

### 3.1. Performance of the ML Method

In this section, we show the performance of the proposed methods for calculating the parameters of a GG distribution. For this purpose, we performed 200 synthetic experiments and tested the methods presented in Section 2.2. Concretely, we tested the method of Stacy and Mihram [14], Gomes et al. [16], Noufaily and Jones [17], and our proposed method of Section 2.2.4. We will refer to them as *Stacy*, *Gomes*, *Noufaily,* and *proposed* methods, respectively.

The synthetic data was calculated in the same way as was done in [17]: a set of gamma-distributed random samples are generated by means of the method proposed in [29] and the GG-distributed data are obtained by taking the 1/*p*th power of the samples. The parameters of the GG distribution were also calculated from sets of parameters in a reasonable dynamic range. The scale parameter *a* was set to 1 in all the experiments since this parameter just affects to the scale of the data. Both, the *p* parameter and the *ν* parameter were obtained from random samples of a uniform RV in the interval [0.3,5].

**Figure 1:**
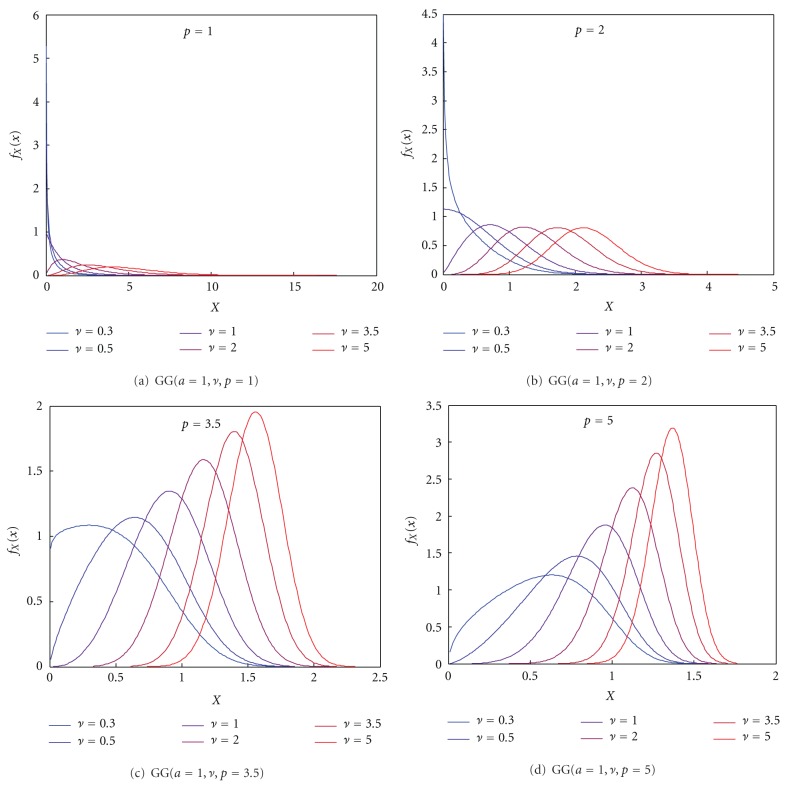
Some examples of the GG distribution for the parameters of the synthetic dataset.

We choose this interval since lower values than 0.3 make the distribution to take values that tend to infinity as *ν* get closer to 0. This is an unrealistic situation when real images are considered. Additionally, when *p* takes lower values, the tail becomes heavier and the shape of the distribution also becomes unrealistic. These effects are shown in Figure 1, where some examples of the PDFs of the GG distribution are depicted.

The number of iterations for the proposed method and for the method of [17] was set to 100 and the tolerance function to 10^−8^. The number of bins where the *χ* ^2^-test was performed in the method of [16] was 150 and the number of samples per experiment was 10^4^. The comparisons of the methods were performed by comparing the goodness-to-fit of each distribution by means of two different measures: Kullback-Leibler divergence (KL) and Kolmogorov-Smirnov (KS) statistic. The former is a nonsymmetric measure of the difference between two probability distributions defined as

(59) $$\begin{array}{c} D_{\text{KL}}\left(p_{n},f_{X}\right)=\sum_{i=1}^{N}p_{n}\left(i\right)\log\frac{p_{n}\left(i\right)}{f_{X}\left(i\right)}, \end{array}$$

where *p*_*n*_ is the empirical PDF estimate and *f*_*X*_ is the theoretical distribution (the GG distribution). For the empirical estimate of the PDF, the number of bins of the histogram was set to 150.

The Kolmogorov-Smirnov statistic is the uniform norm of the cumulative distribution function (CDF), defined as

(60) $$\begin{array}{c} D_{\text{KS}}=\sup\left|\hat{F}\left(i\right)-F_{X}\left(i\right)\right|, \end{array}$$

where $\hat{F}$ is the empirical CDF of data and *F*_*X*_ the theoretical CDF. The KS measure was chosen since it does not depend on the PDF estimate and can be calculated with a few number of samples. Additionally, the Glivenko-Cantelli theorem states that if the samples are drawn from distribution FX, then *D*_KS_ converges to 0 almost surely [30].

**Figure 2:**
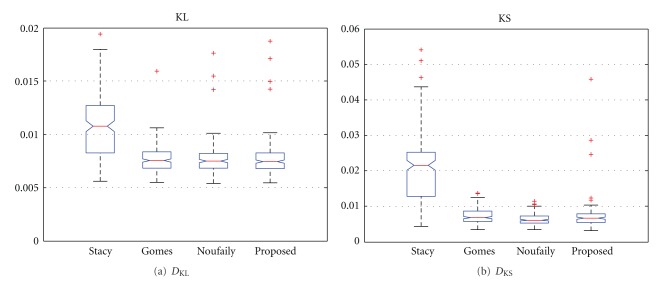
Results for *D*_KL_ and *D*_KS_ for 10^4^ samples. Methods: Stacy and Mihram [14], Gomes et al. [16], Noufaily and Jones [17], and the proposed one of Section 2.2.4.

In Figure 2, the results for both measures are depicted. It is clear that the moments method of Stacy gives poorer results than the other methods for both measures. This result was expected since the moments method depends on moments of third-order, so the variance of the estimates becomes higher. The rest of the methods performed well for both measures. In the case of the *D*_KL_, they fit practically the same while, in the case of *D*_KS_, there are some better results for the method of Noufaily and the proposed one. This is the effect of the approximation of the PDF for the *χ* ^2^ test performed by the method of Gomes: it calculates the best set of parameters for an approximation of the empirical PDF which depends on the number of bins and the number of samples of the dataset. So, as the number of samples is reduced or the number of bins is reduced, the estimate becomes worse.

**Figure 3:**
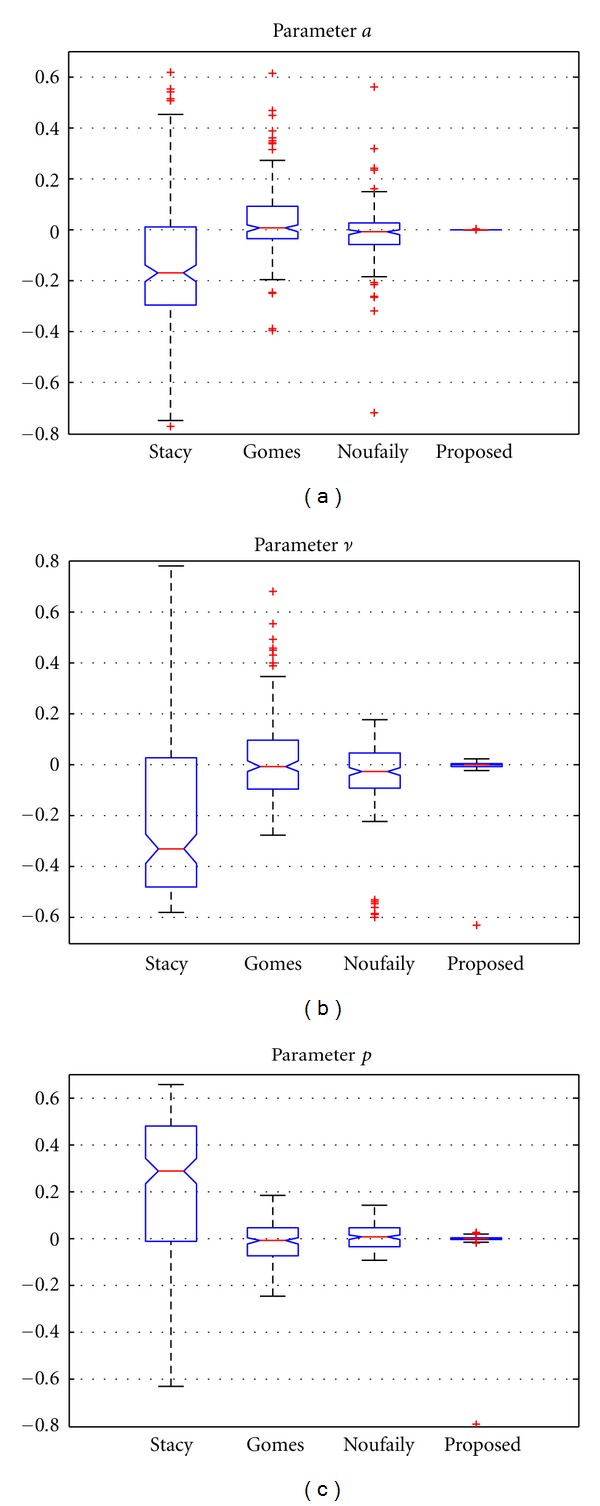
Results for the relative error of the estimates for 10^4^ samples. Methods: Stacy and Mihram [14], Gomes et al. [16], Noufaily and Jones [17], and the proposed one of Section 2.2.4.

In order to see the effect of this, we also show in Figure 3 the relative error of the estimates for all the methods (the relative error of an estimate $\hat{\theta }$ is calculated as $\epsilon_{\mathrm{rel}}=||\theta -\hat{\theta }||/\theta$, while the absolute error is $\epsilon_{\text{abs}}=||\theta -\hat{\theta }||$). In the figure, the whiskers show the dynamic range of the data which is not considered an outlier. So, though the method of Gomes provides good fitting, the variance of the estimates is higher than the method of Noufaily and the proposed one. At first sight, the results of Figure 3 demonstrate the better performance of the proposed method in terms of variance of the ML estimates with no appreciable bias in the estimates.

**Figure 4:**
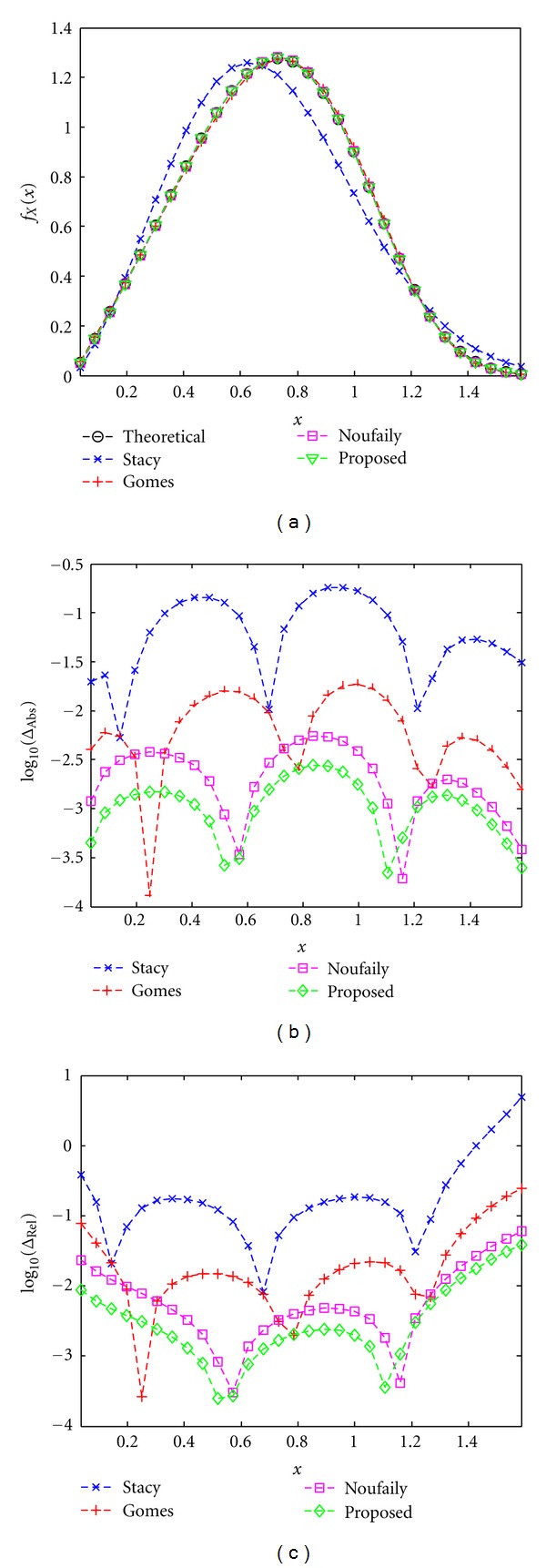
Example of the fitting performance for 10^4^ samples. Methods: Stacy and Mihram [14], Gomes et al. [16], Noufaily and Jones [17], and the proposed one of Section 2.2.4. (a) Probability Density Functions, (b) Absolute error of the PDFs, and (c) Relative error of the PDFs.

An example of the fitting performance of the methods is shown in Figure 4 where the PDFs obtained with the methods are depicted as well as the absolute error and the relative error of the PDFs.

**Figure 5:**
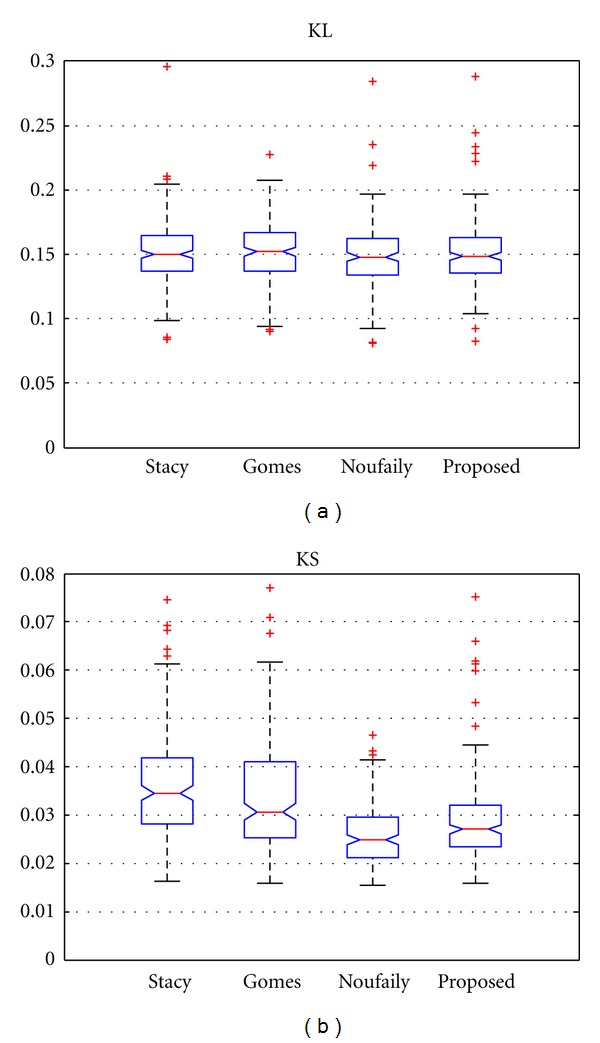
Results for *D*_KL_ and *D*_KS_ for 500 samples. Methods: Stacy and Mihram [14], Gomes et al. [16], Noufaily and Jones [17], and the proposed one of Section 2.2.4.

**Figure 6:**
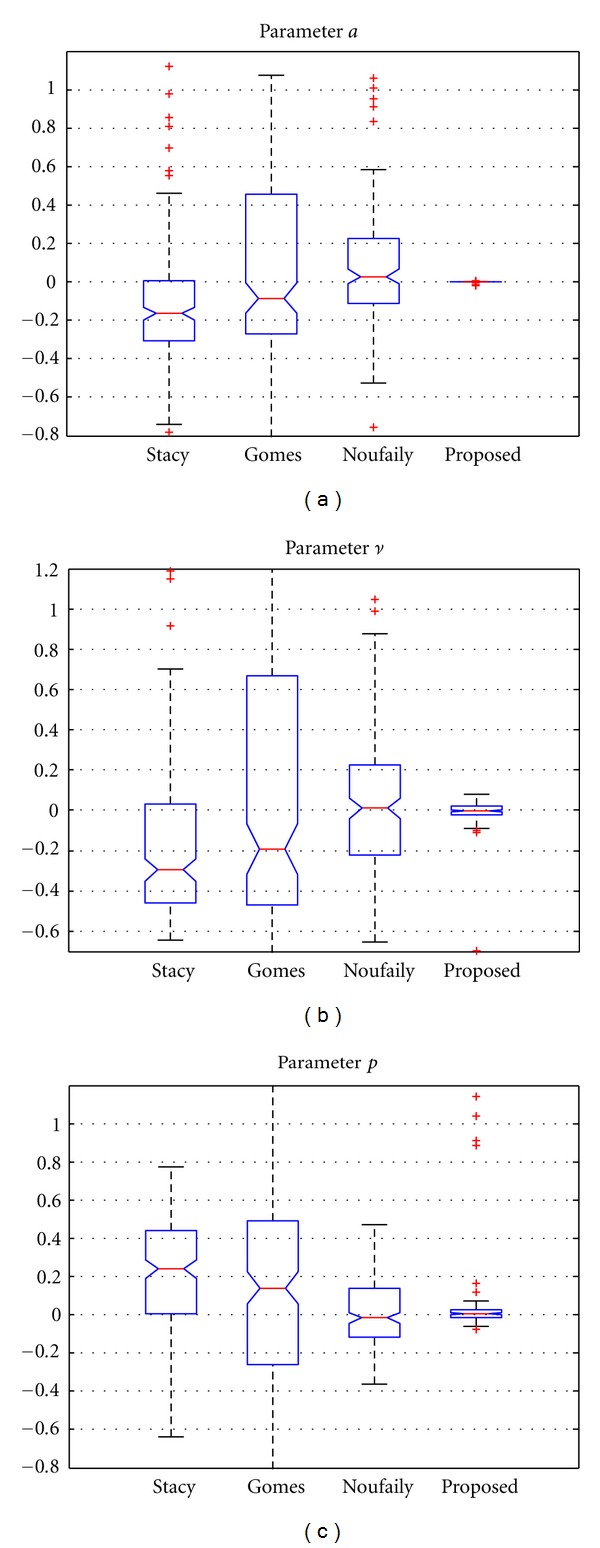
Results for the relative error of the estimates for 500 samples. Methods: Stacy and Mihram [14], Gomes et al. [16], Noufaily and Jones [17], and the proposed one of Section 2.2.4.

Following, we analyze the dependence of the estimates with the number of samples. The same experiment is repeated considering 500 samples. The results of both goodness-to-fit measures are shown in Figure 5, and the relative errors of the estimates are depicted in Figure 6. The performance for the *D*_KL_ measure is similar for all the methods. However, note that the value is considerably higher than the obtained for the case of 10^4^ samples, this effect is caused by the difficulties of estimating the PDF with so few samples. Since the Gomes algorithm is based on the *χ* ^2^ test, it is expected that its performance decreases and the variance of the parameter estimates increases. In the case of the *D*_KS_ measure, the performance of all methods is comparatively equal to the case of 10^4^ samples but a higher variability is observed in the Gomes method due to the sensitivity to the number of samples.

The better performance of Noufaily and the proposed methods are seen in Figure 6 where the variability of the Noufaily method did not increase dramatically as the Gomes method did. The proposed method also presented a very low variance of the parameter estimates with no appreciable bias. In the light of these results, we can conclude that the proposed method is robust with respect to the number of samples and it does not introduce any appreciable bias in the parameter estimates. The goodness-of-fit performance of both the Noufaily and the proposed method are similar, though the estimates are more accurate with the proposed method. This can be due to the better convergence of the Nelder-Mead method than the algorithm of the Noufaily method.

### 3.2. Tissue Characterization in Real US Images

In this section, we test the performance of the GG distribution for characterizing tissues of real images. For this purpose, we used a set of 518 real US images (584 × 145, 8 bits) obtained from 3 human subjects by means of a clinical machine GE Vivid 7 echographic system (GE Vingmed Ultrasound AS, Horten, Norway). The images were acquired before the Cartesian rearrangement. The image collection was supervised by specialists Marta Sitges and Etelvino Silva (Hospital Clinic IDIBAPS Universitat de Barcelona, Spain). The subjects were volunteers for a study of the reconstruction process of ultrasonic images. The acquisition was done in the Hospital Clinic of Barcelona with its approval. The images were provided by Nicolas Duchateau (CISTIB-Universitat Pompeu Fabra, Ciber-BBN, Barcelona, Spain) and Bart Bijnens (Instituco Catalana de Recerca i Estudis Avan cats (ICREA), Spain).

**Figure 7:**
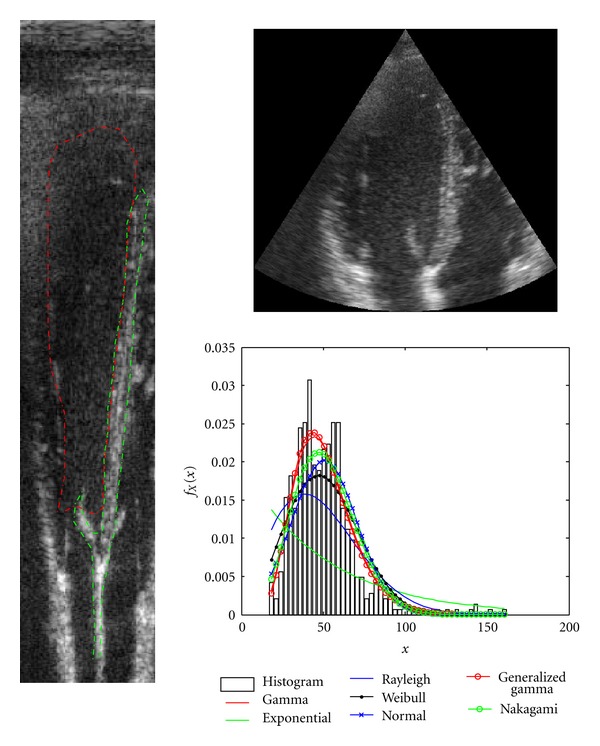
Example of an image of the data set. The red contour is the segmented areas of blood which are considered in the study, while the green contour is the segmented areas of tissue. The intersection of both regions was rejected in the study.

In Figure 7, an example of a real US images is shown with its Cartesian rearrangement. The red contour is the segmented areas of blood which are considered in the study, while the green contour is the segmented areas of tissue. The intersection of both regions was rejected in the study.

Additionally, the histogram of the image was depicted for the blood region as well as the fitted distributions most commonly used to characterize tissue. From the whole data set, a total number of 3185 regions were segmented for myocardial tissue while 1960 were segmented as blood. The sizes of regions vary depending on the tissue. However, it is high enough to provide a good estimate of the parameters. For instance, the segmented region of Figure 7 has 18250 samples for blood and 5529 for tissue.

In the case of Figure 7, the lower value of the histogram shown is 19 since the intensity values in the blood area were in the interval [19,156]. The number of bins used for the representation of the histogram was set to 20 equally spaced in that interval.

**Figure 8:**
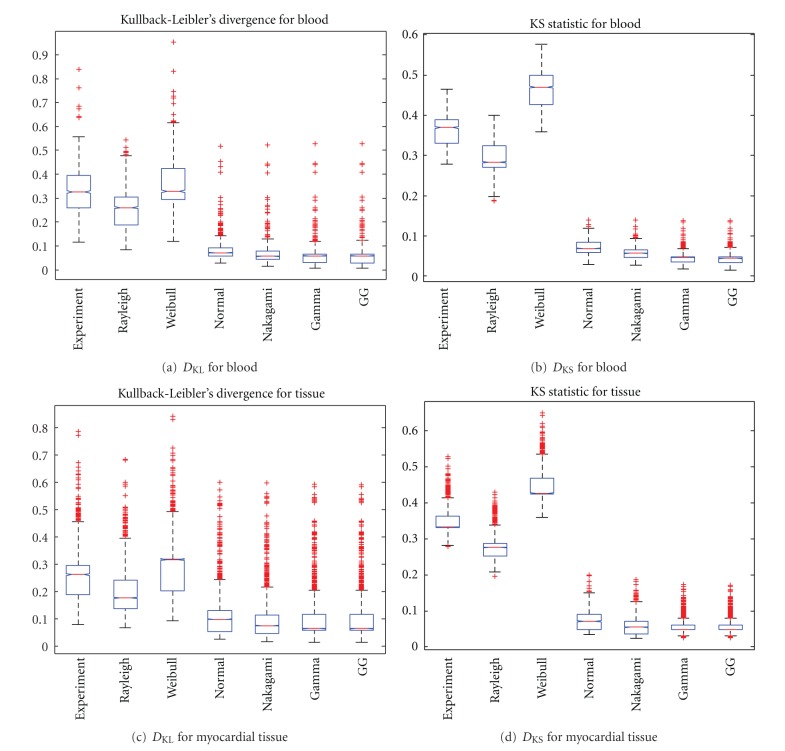
Results for the relative error of the estimates for 500 samples. Methods: Stacy Stacy and Mihram [14], Gomes et al. [16], Noufaily and Jones [17], and the proposed one of Section 2.2.4.

The performance of the GG distributions was tested by estimating the PDFs for both tissue classes (myocardial tissue and blood) for the following distributions: Exponential, Rayleigh, Weibull, Normal, Nakagami, Gamma, and GG. The PDFs were compared by means of both the *D*_KL_ and the *D*_KS_ measures. The results of the comparison are depicted in Figure 8 where the better performance of the Gamma, Nakagami, and GG becomes clear. In order to see whether these measures are statistically significant, we carried out a Welch *t*-test for the Gamma, Nakagami, and GG distributions for the *D*_KS_ measures. This test was chosen since no equal variance should be assumed and the *D*_KS_ since it does not depend on the empirical PDF estimate but just on the samples. The assumed hypothesis *H*_0_ is that “both distributions have the same mean,” *H*_1_ indicates that the null hypothesis can be rejected at a 5% of significance level.

The results are shown in Tables 1 and 2. Note that all the null hypothesis were rejected but just one: myocardial tissue. In that case, the difference of the mean value of the Gamma and the GG is not statistically significant. The mean values of the *D*_KS_ are represented in Table 3 where the lower mean value of the GG for both tissues can be appreciated. The results of the *t*-test of Tables 1 and 2, and the lower mean values of the *D*_KS_ evidence the better performance of the GG than the rest of the distributions, with the exception of the myocardial tissue, where a Gamma distribution offers the same performance.

### 3.3. Performance of the GGMM Methods

In this section, we test the performance of the proposed GGMM methods in three different scenarios. First, we test the necessity of using more than a simple GG for describing tissues with an increasing echolucent response of the effective scatterers. The case of a variation of the number of effective scatterers is also considered. This behavior can be found in structures with an increasing deterministic response that changes the speckle nature from fully formed speckle to fully resolved speckle. The variation of the number of effective scatterers can be found in structures which change their scattering cross-section.

In order to simulate B-mode US images, we followed the same methodology proposed in [9]. This method scans an image and records the data in a matrix which is corrupted by means of the speckle formation model of (1) where the tissue is modeled as a collection of scatters of size comparable to the wavelength. The speckle pattern is obtained by means of a random walk which does not assume any statistical distribution in order to avoid any bias of the results. The Cartesian arrangement is obtained by means of linear interpolation of the corrupted samples.

**Figure 9:**
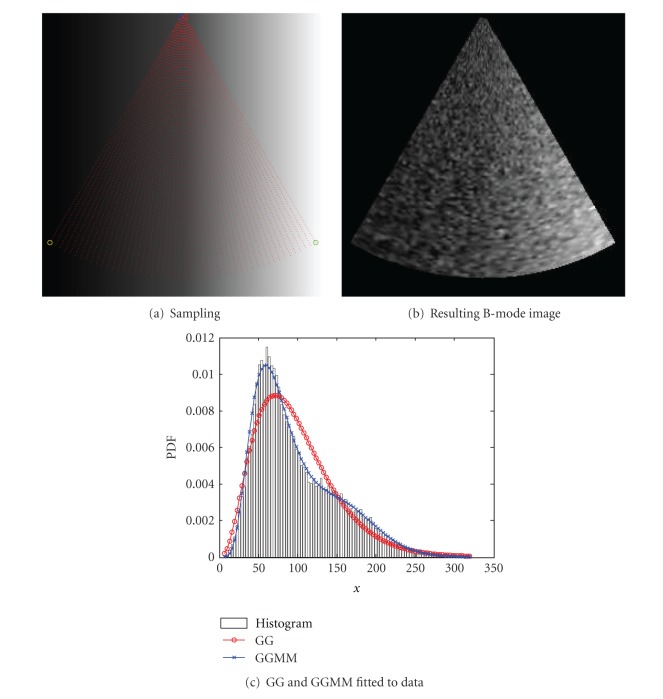
Simulation of spatial variant echolucent response of tissue. (a) Sampling of an increasing echolucent tissue. (b) Resulting B-mode image obtained by corrupting the samples by a random walk process of 20 scatterers per resolution cell, in order to simulate a fully formed speckle in regions with low echolucent response and fully resolved speckle in regions with high echolucent response. (c) Histogram of (b) and (a) GG and GGMM with 2 components.

As a first example, we simulate an increasing echolucent tissue which varies its intensity from 0 to 255 from left to right. The sampling process and the resulting B-mode image are shown in Figures 9(a)-9(b). The number of samples were set to 50 angular samples and 100 radial samples, represented as red points. The amplitude of each scatterer is defined as a Normal distributed RV with zero mean and *σ* = 8. Note that, along with the variation of intensities from left to right, a specular component of the speckle will appear. The number of scatterers was set 20 in order to simulate fully formed speckle in regions with low echolucent response and fully resolved speckle in regions with high echolucent response. The resulting B-mode image is represented in Figure 9(b).

The fitted GG and GGMM with 2 components depicted in Figure 9(c) show that one simple GG fails to model the probabilistic behavior of a spatially variant echolucent tissue, while a GGMM with 2 components properly describes the echolucent variation.

**Figure 10:**
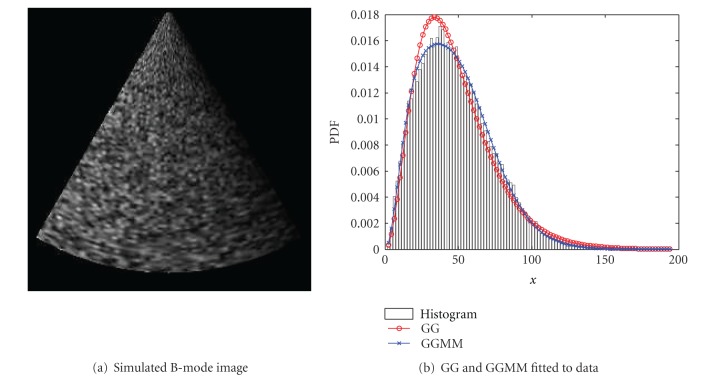
Simulation of spatial variant density of scatterers. The number of scatterers per resolution cell decreases from left to right in order to simulate fully formed speckle in regions with low density and partially resolved speckle in regions with high density.

As an additional experiment, in Figure 10, we represent the spatial variation of the number of effective scatterers. The simulation was performed with the same sampling parameters as was done in the previous experiment. In this case, the echolucid response was set to be homogeneous with no deterministic component. Thus, the nature of the speckle changes from fully formed speckle to partially formed speckle. The number of scatterers decreases from left to right from 256 to 1. The amplitude of each scatterer is defined as a Normal distributed RV with zero mean and *σ* = 8.

The speckle PDF in this case becomes more impulsive in areas with more effective scatterers (left part of Figure 10(a)), this behavior is observed in a lower decay of the tail. Both the simple GG and GGMM with 2 components were calculated from the data and are depicted in Figure 10(b). In that figure, the fitting of a simple GG clearly shows that one component does not suffice to describe a spatial variation of number of effective scatterers.

**Figure 11:**
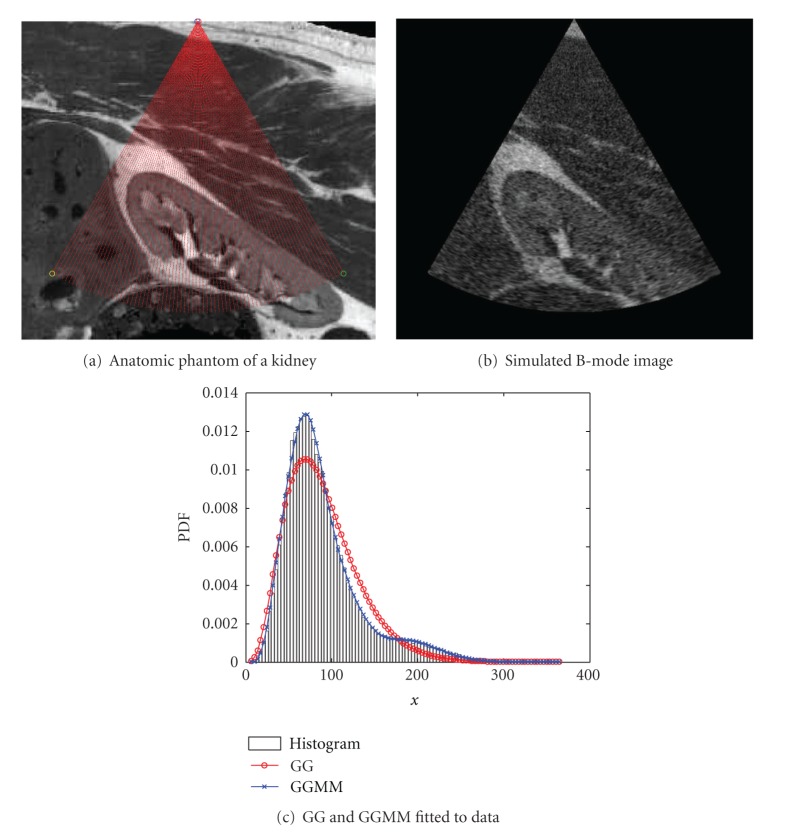
Simulation of an anatomic phantom of a kidney scan.

**Figure 12:**
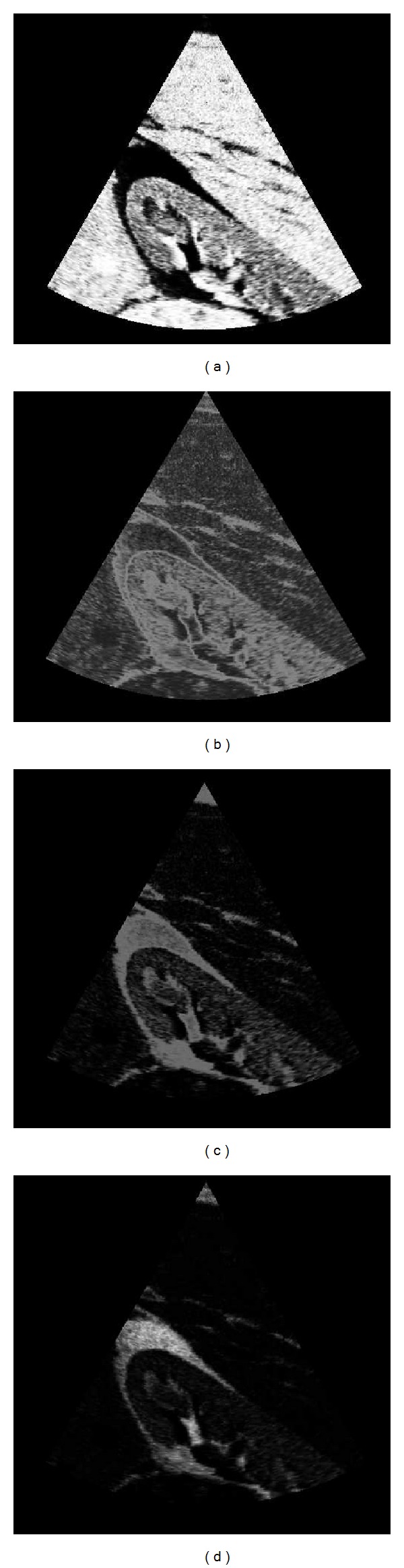
Probability of belonging to each component of the GGMM fitted to the image in Figure 11(b). The components are sorted in increasing mean value.

In the last synthetic experiment for testing the necessity of GGMM, we simulate an anatomic phantom of a kidney scan. For this purpose, we used the artificial kidney scan proposed by Jensen [31]. The image can be downloaded from the Field II website (http://field-ii.dk/). The sampling of the kidney and the resulting B-mode image are represented in Figure 11. In this case, a GGMM with 4 components was used to fit the PDF of the image. The probability of belonging to each component is represented in Figure 12 where the differentiation of tissues can be easily observed. In this case, a lower number of components fail to describe the kidney and the surrounding tissue which have a similar speckle response.

For testing the performance of the proposed GGMM methods with real data, we use the same data set used in the previous section. The number of components is set to two: blood and myocardial tissue. In order to compare the performance of the GGMM methods, we also fit a Gamma Mixture Model and a Nakagami Mixture model to the data [3, 32, 33]. Both the *D*_KL_ and the *D*_KS_ where calculated for the mixture models in each image. The number of iterations for each mixture model was set to 100 and the tolerance to 10^−8^.

**Figure 13:**
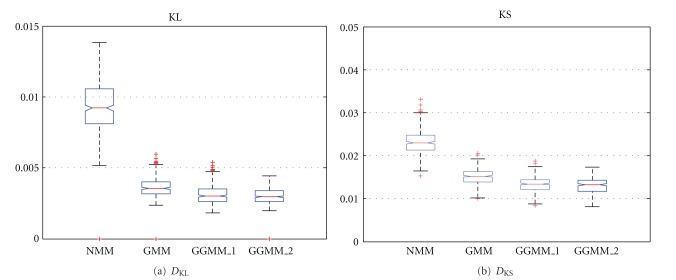
Results for *D*_KL_ and *D*_KS_ of the Mixture Models: GGMM_1_, GGMM_2_, GMM and NMM.

The lower values of *D*_KL_ and *D*_KS_ shown in Figure 13 evidences the better characterization of the GGMM when compared to the NMM or the GMM. These results were expected due to the results of the previous section. Again, the *t*-tests were performed to the *D*_KS_ measure of the data. All the mixtures were statistically different with the exception of the GGMM_1_ and GGMM_2_. In that case, a *P* value of 0.4906 was obtained. These results show once more that the GG can characterize better than other commonly accepted distributions and the differences are significant.

### 3.4. Potential Applications of the GGMM

A proper characterization of the speckle by means of suitable distributions can be used to guide segmentation algorithms as the one in [3]. The parameters of the mixture model can be used as features for developing a classifier as was done in [4]. Furthermore, some filters use the probability of belonging to each tissue class. As an example of the performance of the GGMM, we show some results of the Probabilistic-Driven Oriented Speckle Reducing Anisotropic Diffusion (POSRAD) [8].

This last filter includes the probability of belonging each tissue class and adapts the diffusion tensor. Concretely, it calculates the structure tensor of the posterior probability and detects the most probable edges of the image. This information is used to define the diffusion tensor which provides a better behavior in the boundaries of the image.

The structure tensor of the probability density function for each tissue class is calculated as:

(61) Tj(xi)=Gσ∗(∇σp(Zi=j ∣ xi,Θ)·∇σp(Zi=j ∣ xi,Θ)T),

where *G*_*σ*_ is a Gaussian kernel of standard deviation *σ*, and ∇_*σ*_ *p*(*Z*_*i*_ = *j* | *x*_*i*_, Θ) is the gradient of the probability density function for each tissue class filtered with a Gaussian kernel of standard deviation *σ*. Finally, let *λ*_1_^*j*^ ≥ *λ*_2_^*j*^ be the eigenvalues and (**v**_1_^*j*^, **v**_2_^*j*^) their respective eigenvectors. The local orientation of the maximal variation of probability of the class *𝒞*_*j*_ is given by **v**_1_^*j*^, and the local orientation of the minimal variation is given by **v**_2_^*k*^.

Let us consider the following diffusion equation:

(62) u(x,0)=u0∂u∂t=div⁡(D∇u),

where the matrix *D* is the diffusion tensor which can be described by its eigenvectors (**v**_1_, **v**_2_) and eigenvalues *λ*_1_, *λ*_2_.

Given a diffusion tensor, *D*, the diffusion of the intensity values of the image is performed in the direction of eigenvectors with different diffusion coefficients. For each eigenvector, its eigenvalue defines the diffusion coefficient and, thus, an anisotropic diffusion can be achieved.

As an example, when one eigenvalue is equal to 1 and the other one is 0, a complete anisotropic diffusion is obtained, since the intensity values diffuse in the direction of the eigenvector associated to the eigenvalue equal to 1. This would be the desired behavior of a filter in regions where structures must be preserved. When both eigenvalues are equal to 1, the diffusion process becomes isotropic and the intensity levels diffuse equally in all directions. This case would be the desired behavior for homogeneous regions where no structures must be preserved.

The POSRAD philosophy makes use of the structure tensors determined out of the probability maps to obtain the most probable structures. In that case, the diffusion filter should be anisotropic. When no probable structures are detected, the diffusion should be isotropic.

Since we have *J* structure tensors (each tissue class with probability density function), we choose the eigenbase of the structure tensor with maximal *λ*_1_^*j*^: $\hat{j}={\arg\;\max}_{j}(\lambda_{1}^{j})$. This base gives the orientation of the maximal variation of probability among all the classes.

The interpretation of this choice is that we choose as boundary the one with the maximal gradient of the probability density function over all tissue classes. This way, the most probable boundary is preserved in the filtering process. In the basis of $T_{\hat{j}}$, namely, (**e**_1_, **e**_2_), the diffusion matrix *D* is defined as

(63) $$\begin{array}{c} D=E\left(\begin{array}{cc} \lambda_{1} & 0\\ 0 & \lambda_{2} \end{array}\right)E^{T}, \end{array}$$

where

(64) λ1=1−||∇e1,σp(Zi=j ∣ xi,Θ)||2λ2=1,

and ||·||_2_ is the 2-norm, ∇_**e**_*i*_,*σ*_ is the directional derivative in the direction **e**_*i*_ filtered with a Gaussian kernel with a standard deviation *σ*, and *E* is the matrix whose columns are the eigenvectors (**e**_1_, **e**_2_). This definition performs a diffusion filtering in the direction of the minimal variation of probability (**e**_2_) while preserves the maximal variation of probability since ||∇_**e**_1_,*σ*_ *p*(*Z*_*i*_=*j*∣*x*_*i*_,Θ)||_2_ will have a value closed to 1. Note that the discrete approximations of ||∇_**e**_1_,*σ*_ *p*(*Z*_*i*_=*j*∣*x*_*i*_,Θ)||_2_ is bounded in [0,1], thus *λ*_1_ ∈ [0,1].

**Figure 14:**
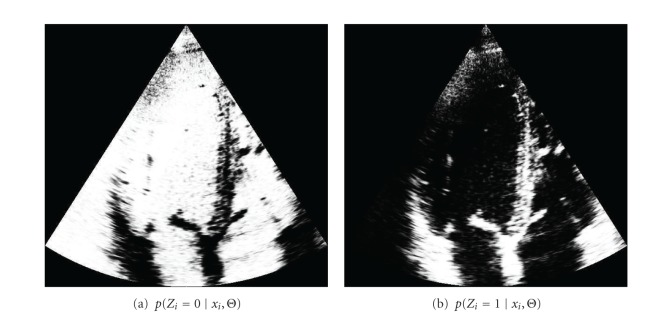
Probability of belonging to each tissue class, where the class 0 describes the blood and the class 1 describes the myocardial tissue.

**Figure 15:**
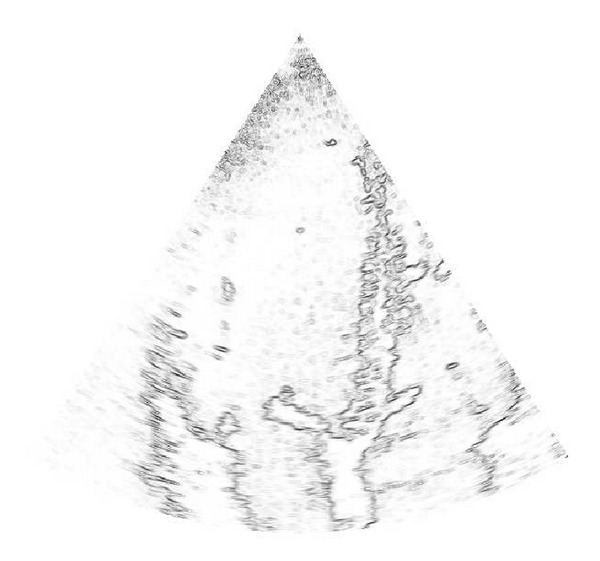
Anisotropic behavior of the filter. The most probably edges of the image are described by the lower values of *λ*_1_ ∈ [0,1].

In Figure 14, we show the probability of belonging to each tissue class, *p*(*Z*_*i*_ = *j* | *x*_*i*_, Θ), provided by the GGMM method (the GGMM_2_ was used for this example). All the figures of the example are represented in their Cartesian arrangement in order to ease visualization of fine structures. Note that the structures are clearly identified by each posterior probability of each tissue class and the filter can perform an efficient anisotropic diffusion. To see this, in Figure 15, we represent *λ*_1_, which describes the anisotropic behavior of the filter. When *λ*_1_ = 1, the filter acts like a conventional isotropic filter, whereas the pure anisotropic behavior is carried out when *λ*_1_ = 0.

**Figure 16:**
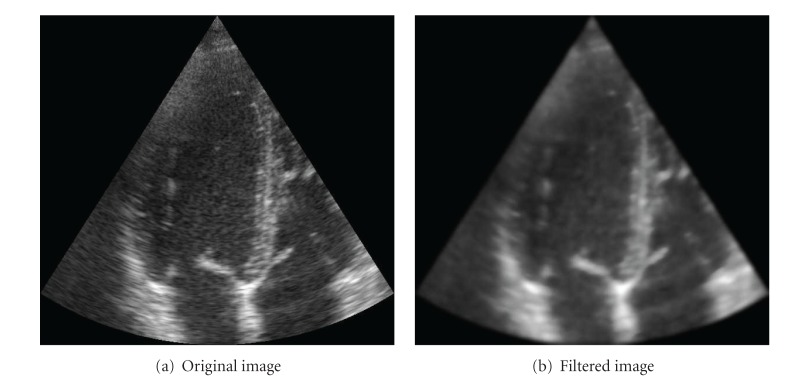
Results of the POSRAD filter. The anisotropic behavior of the filter is appreciated in the preserved details of the myocardial tissues.

Finally, the resulting image after 40 iterations is depicted in Figure 16 in comparison to the original one.

**Figure 17:**
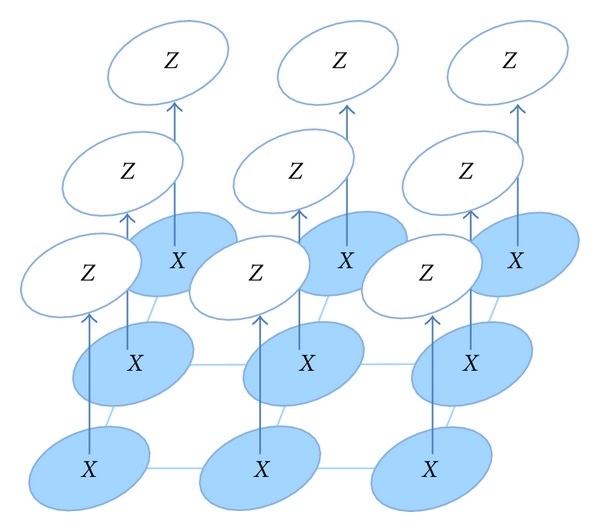
Undirected graph. The nodes represent a random variable *X* and the edges' relationships between nodes. Each random variable can be classified as a tissue class *J*.

As a final application of the GGMM, one can make use of the pixel-wise probability of belonging to each tissue class to obtain a spatially coherent probability by introducing an undirected graph where the nodes (each pixel of the image) represent a random variable and the edges of the graph represent the relationships between nodes as it is represented in Figure 17. The problem is reduced to find the labels for each node by taking into account the relationships between nodes of the local neighborhood (the Markov property is assumed). This problem, though is intractable in terms of direct probabilistic inference, can be solved by means of the Loopy Belief Propagation (LBP) algorithm introduced by Pear in [34]. This algorithm performs approximate inference of a graphical model. Although LBP does not guarantee to converge due to the presence of loops in the graph, however it has shown good experimental results and is commonly used [35].

In the end, the problem is faced as a discrete MRF where the labels, *Z*, are each tissue class and the nodes are the pixels of the image. The energy to be minimized by the LBP method can be defined as

(65) $$\begin{array}{c} V\left(Z\right)=\sum_{i=1}^{N}V_{1}\left(Z_{i}\right)+\sum_{k\in \eta \left(i\right)}V_{2}\left(Z_{i},Z_{k}\right), \end{array}$$

where *η*(*i*) is the neighborhood of the *i*th node, *V*_1_(*Z*_*i*_) = −log⁡*p*(*Z*_*i*_ = *jx*_*i*_, Θ), and

(66) $$\begin{array}{c} V_{2}\left(Z_{i},Z_{k}\right)=-\sum_{k\in \eta \left(i\right)}\log p\left(Z_{k}=z_{i}\;|\;x_{k},\Theta \right). \end{array}$$

**Figure 18:**
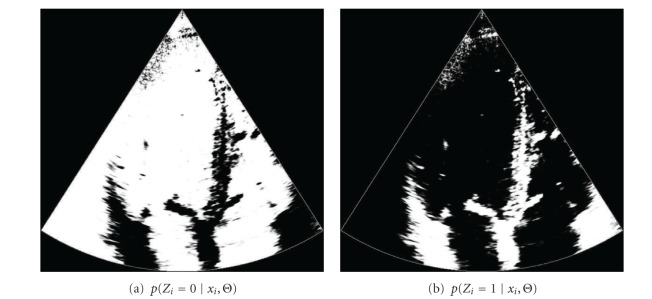
Probability of belonging to each tissue class after the LBP, where the class 0 describes the blood and the class 1 describes the myocardial tissue.

The output of the LBP is a belief of node *i* belongs to class *Z* = *j*. Thus, the probability with spatial coherence can be directly obtained from the outputs of the LBP algorithm. In Figure 18, the probability of each tissue class when the spatial coherence is introduced.

**Figure 19:**
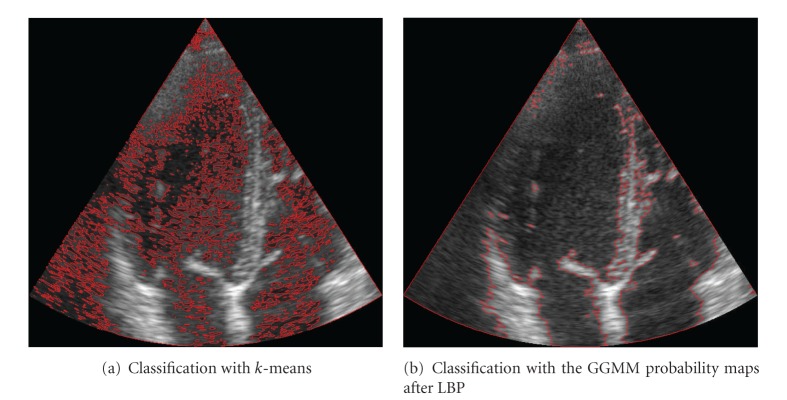
Simple example of the valuable information of the posterior probability obtained from the GGMM with spatial coherence.

These coherent probability maps can be of help for classifying purposes or as prior information for segmentation algorithms. The valuable information that they provide can be seen in a simple experiment in which we consider the classification of two tissues (blood and myocardial tissue) and we compare the results with the *k*-means algorithm applied to the original image and a simple classifier consisting of assigning the class with maximum posterior probability. The results of this example are shown in Figure 19 where the identification of the myocardial tissue is clearly obtained by the posterior probability of the GGMM, whereas the *k*-means method cannot properly define a contour of each tissue.

## 4. Conclusions

Throughout this work we have analyzed the advantages of using a GG distribution for characterizing the speckle in ultrasound images. This distribution offers a suitable way to deal with the impulsive behavior of speckle which causes heavier tails in the distributions. Additionally, the GG is a natural generalization for many distributions commonly used to characterize the speckle: Rayleigh, Gamma, Nakagami, Weibull, Exponential, and Rician [13]. Thus, it has all the advantages of these distributions and avoids some of their generalization problems.

Although some approaches have used this distribution in the literature, the inconveniences of estimating its parameters make this option thorny and not attractive. The problem stems from the inaccurate estimate of the moments method proposed in [14] and the impossibility of obtaining a closed-form ML estimates. Some solutions have been recently proposed such as heuristic methods [16], which are strongly dependent on the number of samples, and iterative methods [17] which depend on the initial condition.

In this work, we have proposed a simple methodology to calculate the ML estimate which offers robust results comparing to the methods in the literature [14, 16, 17]. It is worth to mention that the fundamentals of the ML method of [17] and the proposed one are the same since both try to find the solution of three simultaneous non-linear equations. However, the different optimization technique makes the proposed method more robust. Additionally, the performance for describing speckle was tested in a set of 518 real US images of the heart, in which 3185 regions were manually segmented for myocardial tissue and 1960 for blood.

Results with *t*-tests applied to the *D*_KS_ goodness-of-fit measure demonstrated the better behavior of the GG in most of the cases and in those cases where there were no statistical difference, the other distribution is a particularization of the GG.

The formulation of the proposed method allows to generalize this methodology to a GGMM. These mixture models are of great value due to the different nature of the echogenic response of tissues in the received signal. Two different methods were proposed for the calculation of the GGMM parameters GGMM_1_ and GGMM_2_. Both were developed by applying the EM method in the derivation of the proposed ML method, the optimization technique for GGMM_1_ follows the same approach used for the proposed ML method. The GGMM_2_ method makes use of the optimization technique proposed by [17]. Results when comparing both methods to the GMM and NMM in real images showed the better fitting of the GGMM. No statistical differences were detected between GGMM_1_ and GGMM_2_.

Through this paper we showed the better behavior of the GGMM methods when compared to the RMM, NMM, and GMM for the case of cardiac imaging. The potentials of mixture models have proven a good classification performance in intravascular ultrasonic images for RMM [4]. Additionally, the NMM showed good results for segmentation in carotid arteries [3]. In the case of filtering Cardiac imaging, the mixture models have also shown good results [9].

We think the GGMM methods here proposed can be used with good results in the aforementioned modalities since they generalize the RMM, NMM, and GMM in a natural way and allow to describe heavier tails of the PDFs that the RMM, NMM, and GMM fail to fit. Many other US modalities such as breast, liver, and kidney should be considered. We hope the proposed GGMM methods can encourage future research for tissue characterization in those different US modalities.

Finally, we want to recall that the potential applications of GGMM do not confine to those proposed in this paper. We hope the results of this work can revive the use of the GG distribution and its extension, the GGMM, in many other areas.

## Figures and Tables

**Algorithm 1:**
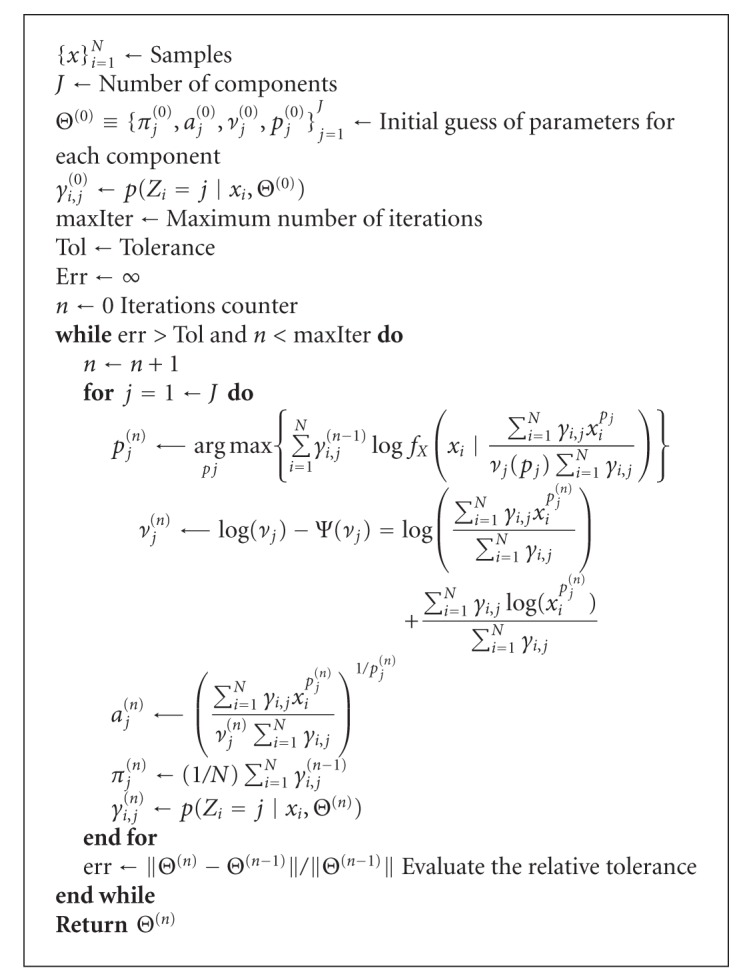
Implementation of the GGMM_1_ method.

**Algorithm 2:**
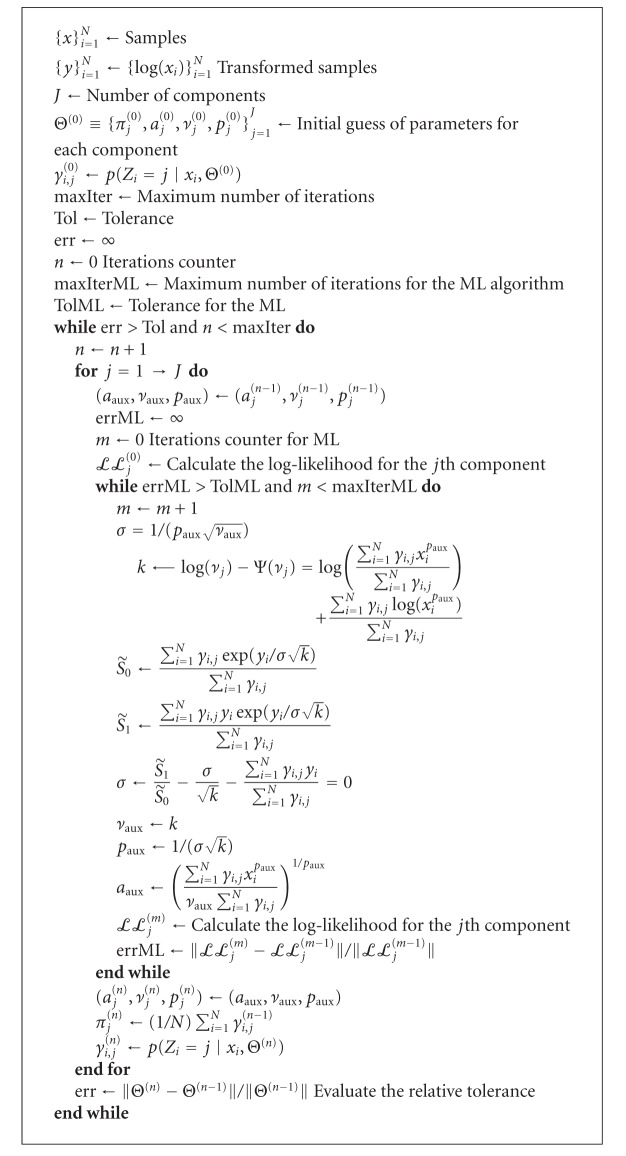
Implementation of the GGMM_2_ method.

**Table 1:** Results of the *t*-test for blood.

Blood	*P* value	Hypothesis
Nakagami versus Gamma	<10^−15^	*H*_1_
Gamma versus GG	3.38 · 10^−7^	*H*_1_
Nakagami versus GG	<10^−15^	*H*_1_

**Table 2:** Results of the *t*-test for myocardial tissue.

Myocardial tissue	*P* value	Hypothesis
Nakagami versus Gamma	3.24 · 10^−4^	*H*_1_
Gamma versus GG	0.96	*H*_0_
Nakagami versus GG	2.74 · 10^−4^	*H*_1_

**Table 3:** Mean values for *D*_KS_.

	Nakagami	Gamma	Generalized Gamma
Blood	5.5626 · 10^−2^	4.4970 · 10^−2^	4.2860 · 10^−2^
Myocardial	5.7711 · 10^−2^	5.5665 · 10^−2^	5.5644 · 10^−2^
